# Peritumoral brain zone in glioblastoma: biological, clinical and mechanical features

**DOI:** 10.3389/fimmu.2024.1347877

**Published:** 2024-02-29

**Authors:** Alberto Ballestín, Daniele Armocida, Valentino Ribecco, Giorgio Seano

**Affiliations:** ^1^ Tumor Microenvironment Laboratory, UMR3347 CNRS/U1021 INSERM, Institut Curie, Orsay, France; ^2^ Human Neurosciences Department, Neurosurgery Division, Sapienza University, Rome, Italy

**Keywords:** glioblastoma, peritumoral brain zone, recurrence, tumor microenvironment, local therapy, cell plasticity and cell reprogramming, brain tumor, surgical resection

## Abstract

Glioblastoma is a highly aggressive and invasive tumor that affects the central nervous system (CNS). With a five-year survival rate of only 6.9% and a median survival time of eight months, it has the lowest survival rate among CNS tumors. Its treatment consists of surgical resection, subsequent fractionated radiotherapy and concomitant and adjuvant chemotherapy with temozolomide. Despite the implementation of clinical interventions, recurrence is a common occurrence, with over 80% of cases arising at the edge of the resection cavity a few months after treatment. The high recurrence rate and location of glioblastoma indicate the need for a better understanding of the peritumor brain zone (PBZ). In this review, we first describe the main radiological, cellular, molecular and biomechanical tissue features of PBZ; and subsequently, we discuss its current clinical management, potential local therapeutic approaches and future prospects.

## Introduction

Glioblastoma (GB) is the most common malignant primary tumor of the central nervous system (CNS). It represents 50.1% of malignant brain tumors and 14.2% of all brain tumors, with an approximate incidence rate of 3 per 100,000 people and five-year relative survival rate of 6.9% (1). The current treatment consists of surgery followed by radiotherapy with concurrent and adjuvant temozolomide (2, 3). However, despite this aggressive standard of care, the prognosis for these patients is poor and the median survival time after diagnosis is 8 months (1).

Histological analysis has been the established approach for GB diagnosis, but nowadays, this main approach is supported by novel diagnostic technologies, such as DNA methylome profiling and advances in molecular diagnostics, especially at the single-cell level, which have reshaped CNS tumor classification. According to the new classification, GB is classified as isocitrate dehydrogenase (IDH) wild-type, representing the most aggressive form of diffuse gliomas (4). Genetic and epigenetic profiling has proven its molecular heterogeneity at inter- and intra-tumor levels. First, bulk RNA-seq studies showed inter-tumor heterogeneity defining three main GB subtypes: proneural (PN), classical (CL), and mesenchymal (MES) (5). Later, scRNA-seq emerged to characterize intratumor heterogeneity, and genetic alterations in CDK4, PDGFRA, EGFR, and NF1 can favor four different cellular states that may coexist in the same tumor and recapitulate neural-progenitor-like (NPC-like), oligodendrocyte-progenitor-like (OPC-like), astrocyte-like (AC-like), and mesenchymal-like (MES-like) cellular states (6). The plasticity between these cell states allows tumor cells to adapt to different treatments, thus evading or resisting the current therapeutic approaches (7). Furthermore, intrinsic phenotypic adaptation also exists in the microenvironment of the tumor core and peritumor brain zone (PBZ), which adds even more complexity to our understanding of this dynamic biological disease (8, 9).

However, despite significant progress in understanding the biology of GB, it remains incurable and there have been no significant therapeutic advances over the past two decades (10). Many biological factors have contributed to this relative lack of progress. The current therapeutic approach involves micro-neurosurgical resection, followed by chemoradiotherapy. The objective of surgical removal is gross total resection (GTR) of the tumor, which has a major effect on the overall survival (OS), progression-free survival (PFS), and quality of life (QoL) of patients (11–13). A balance between surgical cytoreduction and the preservation of neurological function is required to achieve the true benefit of neurosurgical resection. Despite significant technical improvements and contrast agents for enhanced fluorescence-guided surgery, macroscopic GTR is achieved in only 50% of cases (14–17). This is mainly due to the involvement of functional areas, which makes it impossible to perform tumor resection with safe margins without the risk of neurological impairment (12). Furthermore, GB is an infiltrative disease that can extend beyond the contrast-enhancing portion (2, 18). The extent of resection (EOR) is evaluated using early postoperative magnetic resonance imaging (MRI) performed within 72 hours after tumor resection (3, 11). Despite complete surgical resection of the contrast-enhancing portion of the tumor, chemoradiation is not sufficient to avoid regrowth of resistant cells that were not removed by surgery. Therefore, in most cases, recurrence occurs at the margin of the resection cavity, where substantial invading tumor cells are located (19, 20). This poorly characterized area, whose microenvironment interacts with the tumor recurrence-initiating cells, is called the PBZ (14, 21–23). There is a need to biologically characterize the tissues surrounding GB tumors (24, 25); however, preclinical and clinical multicenter studies are lacking in this regard.

The PBZ refers to an area of several centimeters around the tumor that contains specific molecular, radiological, and cellular alterations that are not limited to the immediate area surrounding the contrast-enhanced tumor, which promotes GB cell proliferation, invasion, and recurrence (26, 27). The radiological definition of the PBZ corresponds to the brain area surrounding the tumor without contrast enhancement in T1 gadolinium-enhanced MRI (24). A lack of contrast enhancement with gadolinium-based contrast agents during MRI of the PBZ does not necessarily indicate the absence of tumor cells in this area (24). PBZ radiological and macroscopic analyses have revealed that the PBZ resembles normal brain tissue; however, few cellular, molecular or histopathological studies have been performed in this area (14, 28–30). The biological and molecular characteristics of PBZ may be highly relevant as therapeutic targets, accordingly, proper diagnostic approaches for identifying these areas are needed. Therefore, through an examination of the scientific production from various research groups focused on this topic (24, 31), this article provides an in-depth exploration of the cellular spatial heterogeneity within the PBZ of GB and explains the relevance of the tissue mechanics as an influencer of GB biological niches. In addition, we describe the main radiological features of PBZ and discuss the clinical management of it, with novel local therapies, research approaches and treatment perspectives.

## Cell spatial heterogeneity in the PBZ

**Figure 1:**
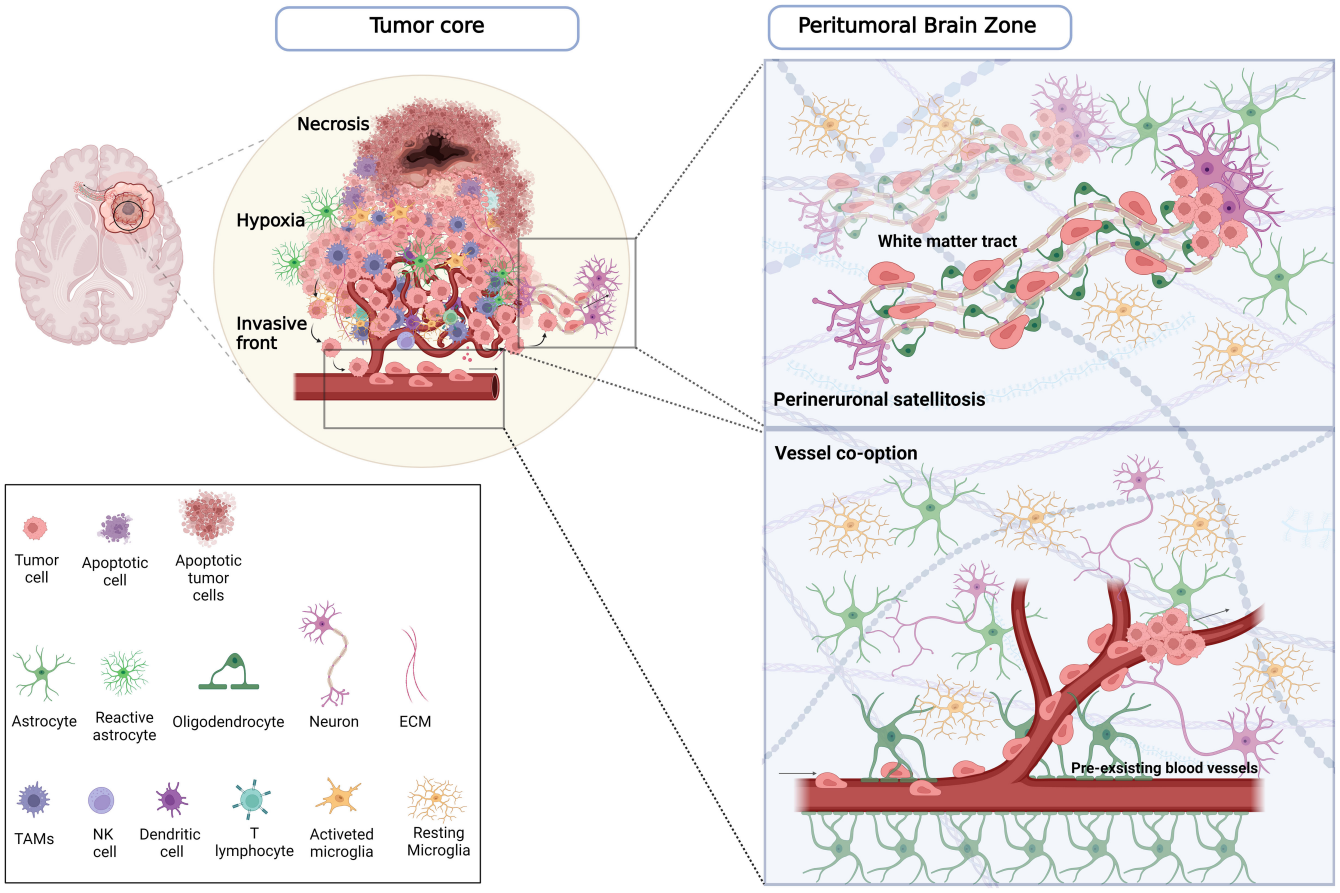
Comprehensive schematic of the distribution of tumoral and non-tumoral cells across the tumor core and PBZ. On the left, the tumor core is characterized in the inner part by a necrotic region in which apoptotic tumor cells and immune cells reside. A hypoxic region encloses the necrotic area surrounded by pseudopalisading cells, which promotes tumor growth, angiogenesis, migration and invasion, and recruits innate immune cells, including macrophages and activated microglia. At the invasive front, endothelial cells, pericytes, activated microglia, reactive astrocytes, and neurons are among the major cell types that make up the GB microenvironment in this region, where glioma cells infiltrate the brain parenchyma in PBZ. On the right, the illustrations of the PBZ, represent the main migration routes by which infiltrating tumor cells invade healthy parenchyma. In the panel below, tumor cells co-opting pre-existing blood vessels outside the tumor core are able to migrate and penetrate the surrounding tissue. In the panel above, the tumor cells, in a process called perineuronal satellitosis, can also take advantage of the nerve fibres and white matter bundles that connect the two hemispheres (corpus callosum), and different brain regions using them as a highway to invade far away from the primary tumor lesion making its surgical resection and treatment almost impossible. Created with www.BioRender.com.

In addition to this vast intertumor heterogeneity, which profoundly affects treatment outcomes and patient prognosis, well-defined intratumor heterogeneity has been reported in the last few years. The histological features exhibit a significant discrepancy between the bulk tumor core and the PBZ surrounding the tumor mass (32) (**Figure 1**). The GB core is distinguished by high cellular proliferation, inflammation, hypoxia, and pseudopalisading areas, i.e. peculiar necrotic zones surrounded by GB cells (31). While the periphery of the GB is primarily composed of brain parenchymal tissue with isolated tumor-infiltrating cells.

### Infiltrating GB cells in PBZ

The presence of GB cells with self-renewal and multi-lineage differentiation properties, called GB stem-like cells (GSCs), has been proposed as the main cause of tumor initiation, growth, and recurrence during GB progression (33). GB mimics developmental-like lineage hierarchies at the cellular level (6, 34). GSCs are at the top of this hierarchy, and are capable of self-renewal and differentiation into non-stem GB cells (33, 35). GSCs with varying stemness have a significant impact on treatment success rates. Different types of GSC niches have been characterized in the tumor core and PBZ, and these distinctions contribute to the progression and therapeutic resistance of GB (36, 37).

According to Neftel et al., GB cells exist in four main cellular states that recapitulate the distinct neural cell types. GB cell lineage progression proceeds towards neural-like fates, such as mesenchymal-like (MES-like), astrocyte-like (AC-like), and neural progenitor-like (NPC-like), or oligodendrocyte progenitor cell (OPC-like) states (6). However, our understanding of lineage evolution in invasive GB cells remains limited. Differences in stemness and molecular characteristics between GB cells isolated from the tumor core and proximal margin regions of patient-derived xenograft models and GB patients have been reported. Anyway, results have been inconsistent and sometimes contradictory because of the heterogeneity in the brain and tumor regions, which highlights the necessity for a more consistent analysis (8, 38–40).

The PBZ is profoundly affected by molecular and metabolic changes of the tumor bulk, making it very different from the normal brain and much closer to the tumor core (41, 42). Therefore, distinguishing GB cells from normal cells in the PBZ is challenging (43), and one of the main problems is the collection of samples from a PBZ area in GB patients compared to a collection of healthy glia or subject to other pathologies (16, 21). Thus, to identify biological markers capable of discriminating and recognizing cancer cells in the PBZ, it is vital to explore the differences between the PBZ and the tumor core.

Different GSC niche localizations across tumor tissues affect their proliferation, self-renewal, molecular subtyping, and radioresistance characteristics (8). The hypercellular hypoxic/peri-necrotic niche is crucial for maintaining the stemness of GSCs while promoting self-renewal, which expands the GSC pool throughout the tumor until it reaches healthy parenchyma and retains its GSC signature (44, 45). Moreover, the peri-necrotic niche in the tumor core is characterized by greater proliferation in contrast to the PBZ, where a more invasive and less proliferating GSCs phenotype is determined (46).

Although a definite distinction in molecular spatial heterogeneity remains unclear, according to Li et al., CD109 (MES marker) and CD133 (PN marker) are mutually exclusive when expressed within a single GSC and their expression appears to indicate a dynamic molecular state (47). The presence of CD133^low^/CD109^high^ GB cells (linked to MES) was associated with the tumor core, whereas that of CD133^high^/CD109^low^ GB cells (linked to PN) was associated with PBZ. However, their expression levels can change after the treatment. These results revealed a significant association between the CD133^low^/CD109^high^ signature and poor prognosis in patients with GB, associated with a substantial transition toward the mesenchymal subtype during recurrence (47). Furthermore, the same group showed that Olig2^high^ GB cells, GSCs typical of PBZ, coexist with CD109^high^ cells, which are typical of tumor core, suggesting that the PBZ and tumor core signature constitute a combination of different GB subtypes. The authors also noted that Olig2 expression was markedly reduced and CD109 expression was higher in MES tumors. When researchers compared the distribution of GB cells from the PBZ and the tumor core in various tumor areas, they discovered that the tumor core was enriched in CD109^high^ cells, whereas the PBZ had a higher concentration of Olig2^high^ GB cells. Additionally, they highlighted a unique marker signature attributable to the tumor core (CD44, MYC, HIF1α, VIM, ANXA1, CDK6, and JAG1) and PBZ (OLIG1, TC2, SRRM2, ERBB3, PHGDH, and RAP1GAP) (8). These gene markers are part of different transcriptional subtypes, implying that the PBZ is a mixture of subtypes. This suggests that beyond the well-known PN-MES axis, there are also spatial implications pointing to a core-to-periphery axis.

The clinical relevance of GSCs that express high levels of CD44 in disease development is not well understood. According to a recent study by Nishikawa et al., higher CD44 expression in the PBZ than in the tumor core was associated with a highly invasive feature, which was correlated with early tumor development and a worse prognosis for survival rates, whereas lower CD44 expression in the PBZ was associated with low invasion and a longer survival rate (48). High CD44 expression is associated with the GB mesenchymal subtype (49), and low survival has been shown to be associated with CD44 expression (50). However, CD44 was preferentially expressed together with CD109 in the tumor core, where a mesenchymal signature in the perinecrotic and pseudopalisading tumor regions was found, compared with the PBZ, where a predominant proneural signature is established (46). Thus, GB cells exhibiting CD44 are not only characterized by a mesenchymal subtype and marked proliferation but are also able to migrate and infiltrate the PBZ, thus causing tumor relapse. Such GB cells behavior may need a phenotypic shift from invasive to proliferative tumor forms and viceversa, in accordance with the ‘Go or Grow’ dichotomy of cancer cells. According to this theory, alterations in the microenvironment, including hypoxia or nutrient depletion, drive a tumor cell to “Go” in search of a more favourable environment and re-establish there, or to “Grow” if the environment provides adequate oxygen and nourishment (51). Hence the possibility, as already proposed by Herrlich et al., that CD44 may exhibit two different functions that encourage the growth or invasion of tumor cells (52). Given this evidence, CD44 could play an essential role in tumor cells adapting to microenvironmental, therapy-induced, oxygen, and nutrient gradient conditions. Hence, a deeper knowledge of its role in GB could guide research towards innovative therapeutic strategies.

### The invasive strategies of GB cells in the brain parenchyma

One of the key causes of GB recurrence and poor prognosis is the infiltration of GB cells into the healthy brain (53). Even with the most advanced imaging tools, detecting solitary cells that have migrated into healthy brain parenchyma is extremely challenging. GB cells can spread widely and even enter the contralateral hemisphere, rendering complete surgical excision of the GB unfeasible (54). GB cells not only move away from hypoxic zones within the tumor core but also have the propensity to infiltrate normal tissues. GB cells are known to penetrate the brain parenchyma via important Scherer pathways, including white matter tracts and blood vessel structures (27, 55). In particular, invasive GB cells prefer to move along myelinated fiber tracts, such as the corpus callosum and internal capsule, meninges, ventricular lining, or on basement membranes found in blood vessels and perivascular regions of the subependymal space (56). The morphology of the brain blood vessels has a diameter ranging from 5 to 50 µm, on which GB cell collections might be concentrated (57), whereas the architecture of the white matter tracts corresponds to high aspect ratio fibers (nanosized diameter and significant length fibers) that prompt cells to detect and stimulate migration (58). These extended, uninterrupted pathways are ideal for encouraging effective cell migration in the PBZ (59). As blood vessels in the perivascular region supply oxygen and nutrients, GB cells are drawn into this area. Indeed, several studies have demonstrated the importance of blood vessels in glioma cell invasion routes (60). Most notably, it has been demonstrated that when GB cells are injected into the brain, more than 85% come in contact with blood vessels (61). Moreover, beyond their function in blood supply, endothelial cell chemoattractants can also encourage the migration of GSCs and invasive GB cells to the perivascular space around blood arteries, enabling them to withstand therapeutic hits, including radiation, facilitating their infiltration into the PBZ (62–64). The perivascular niche allows GB cells to infiltrate healthy brain parenchyma via two distinct mechanisms. A well-known phenomenon that affects nearly all types of tumors is called tumor angiogenesis, which is lead by pro-angiogenic substances such as fibroblast growth factor (FGF), vascular endothelial growth factor (VEGF), angiopoietins, and others synthesised in hypoxic tumor cells (65). At the same time, tumor cells can travel towards and then along pre-existing blood vessels through a process known as vessel co-option, which massively occurs in the PBZ. Vessel co-option (66, 67), refers to the process by which GB cells infiltrate around normal brain vessels to form perivascular cuffs, absorbing the preexisting capillaries into the tumor (68).

On the other hand, white matter, which accounts for approximately 60% of the brain, is the other major route of infiltration (60), and it is composed of tracts or bundles of myelinated axons. Often, GBs originate in white matter and use myelinated fibers as scaffolds for cell migration to expand throughout the brain (69). This includes propagation to the other hemisphere, which only occurs along the corpus callosum’s white matter tracts (60). Surprisingly, very little is known about the cellular and molecular mechanisms underlying white matter invasion. Recently, Brooks et al. identified the white matter as a differentiation niche for GBs with oligodendrocyte lineage competency (70). They found that GB cells that come into contact with the white matter take on the fate of oligodendrocytes, which inhibits their proliferation and invasion. This study emphasizes that differentiation is a reaction to white matter damage caused by tumor infiltration, which creates a feedback loop that suppresses tumor growth via SOX10 making it a potential therapeutic target. Hence, even though invasion along the white matter is a typical infiltration route towards and within the PBZ, myelin damage results in tumor growth suppression, slowing the spread of GB (70).

### Non tumor cells in the PBZ

The tumor ecosystem in the GB consists of neurons, astrocytes, oligodendrocytes, glioma-associated stromal cells, endothelial cells, pericytes, microglia, tumor-infiltrating immune cells – such as tumor-associated macrophages (TAMs) and tumor-infiltrating lymphocytes (TILs) – as well as extracellular matrix (ECM) components, extracellular vesicles, and soluble secreted ECM remodelling enzymes (71). To maintain their growth, GB cells establish multiple communication routes with brain-resident cells via cellular secretion, gap junctions, and tunnelling nanotubes (25, 72, 73). Recently, some of these mechanisms have been identified and used to develop novel therapeutic strategies (74). However, the intricate cell-cell interactions between the GB and stromal cells in the PBZ remain partly unknown. In addition to maintaining brain homeostasis, neurons and astrocytes play a significant role in tumor development and contribute to their survival in the PBZ. They increase Ca2+ activity of the GB cells followed by a shift towards a more MES-like state as a result of plastic changes to surgery, radiotherapy, and chemotherapy, leading to increased resistance (75). However, the role of astrocytes and, to a lesser extent, oligodendrocytes in the GB context and their interactions with tumor cells remain elusive. Although studies on the neural regulation of GB formation have made significant progress, some issues remain unresolved.

### Neuronal cells in PBZ

GB cells can exploit the mechanisms that normally underlie brain development and the plasticity of neural circuits to ensure growth and proliferation. Throughout normal development, electrical activity affects central and peripheral nervous system formation (76). Furthermore, recent studies have revealed that GB cell behavior is influenced by brain activity in a region-specific manner (77). Moreover, glutamate, which causes seizures and cytotoxicity in PBZ networks, is released by GB cells and acts in a paracrine and autocrine manner, promoting the spread of GB cells into the brain parenchyma (78). In addition, the identification of neuronal activity-dependent paracrine signalling of neuroligin-3 (NLGN3) and BDNF led to the first understanding of the mechanisms of activity-regulated paracrine signalling (79, 80). GB cells can also structurally integrate into neural circuits, forming neuron-glioma synapses that facilitate GB invasion within the PBZ of the tumor in a calcium-dependent manner (81). In this study, the authors first examined the direct gap junction-mediated interactions among themselves and with astrocytes to then investigate the tumor network concept in the brain tumor microenvironment. They identified both connected and unconnected GB cell subtypes and showed that the highly connected GB cells, which are present in the tumor core, are more stationary, have a larger density of tumor microtubules (TMs) among cells, and are stable over time. In contrast, the unconnected GB cells present in the PBZ were more invasive and had higher TM turnover. According to Humphries et al., TM turnover is crucial, because it resembles a peculiar searching pattern typical of animals called Levy-like movements (82), which also reflects an optimal search strategy of TM protrusions in the tumor ecosystem to increase their invasiveness (81), reminiscent of the dynamics typical of the migration of immature neurons (83). Moreover, they found that unconnected GB cells in the PBZ were enriched in neuronal and oligodendrocyte precursor-like cell states, and showed neurodevelopmental transcriptional signatures. In contrast, connected GB cells exhibit an MES-like and an injury response transcriptional signature in the tumor core. Additionally, they discovered that higher neural activity in the PBZ resulted in more TM branching and turnover, suggesting that neuronal inputs drive TM dynamics during invasion. Along with these results from gene and pathway analyses, AMPAR gene expression has also been found to be enriched and associated with higher invasiveness at the tumor rim (81).

This novel interplay between neurons and GB cells raises questions about the implications in terms of therapy resistance, as well as the impact that surgery has on tumor recurrence. Moreover, it invites the consideration of how these differently connected regional differences respond differently to surgical and therapeutic interventions.

Lastly, it has been shown that the GB cell network includes cells with rhythmic Ca^2+^ activity that influences cell growth and proliferation in a MAPK and NF-ĸB pathway-dependent manner and is related to the calcium-activated potassium channel KCa3.1. The presence of the channel is related and restricted only to a small group of GB cells with spontaneous and oscillatory activity that communicate and interconnect via gap-junctions with the rest of the GB cell network, and the direct ablation of these “peacemaker cells”, profoundly compromised the Ca^2+^ activity, leading to increased cell deaths (84). Interestingly, the presence of these tumor cells is restricted to the tumor core and is largely absent in the PBZ (84), which, as mentioned previously, is characterized by an unconnected GB cell distribution pattern (81). Thus, even the extent of surgical resection in the non-enhancing fluid-attenuated inversion recovery (FLAIR) zone could affect how quickly the tumor recurs, not only based on how many GB cells are removed but also on how this pattern of connections is perturbed. Hence, in conclusion, therapy should be reconsidered in terms of region-based therapy and flanked by systemic therapy.

### Astrocytes and reactive astrogliosis

Astrocytes are the most common glial cells in the CNS. They carry out crucial tasks related to growth and homeostasis, including maintaining the blood-brain barrier (BBB), storing and supplying energetic substrates to neurons, assisting in the growth of neural cells and synaptogenesis (85). Additionally, astrocytes can regulate microglial activity, attract inflammatory cells to the CNS, and even inhibit neurodegeneration and CNS inflammation through a variety of mechanisms such as neurotoxicity, metabolic cascades, and modulation of microglial activities (86–88).

As a result, it is not surprising that they also react with the GB cells. GB cells successfully co-opt astrocytes to maintain their proliferation, survival, migration, and therapeutic resistance (89). Cancer patients with high reactive astrocyte gene expression levels have a worse prognosis, and tumor cells co-transplanted with astrocytes in mice result in more aggressive tumors (90).

GB cells communicate with astrocytes via the gap junction protein connexin-43 (CX-43), which increases chemotherapy resistance and GB cell proliferation and migration (91, 92). Astrocytes and GB cells express CX-43 via gap junction proteins. The role of astrocytes in the GB microenvironment is partially explained by CX-43-mediated gap junction coupling between GB cells and astrocytes, which alters their phenotype and hence fosters a more pro-invasion environment for GB cells (93, 94).

A subpopulation of astrocytes specifically localized in perivascular areas, where a population of CD44^+^ GB cells also resides, was identified by comparing the gene expression patterns of tumor microenvironment-associated astrocytes in low- and high-grade gliomas. Osteopontin, a CD44 ligand that promotes stemness of GB cells, is highly expressed in astrocytes. The correlation among osteopontin, CD44, and poor OS in patients with GB raises the possibility that perivascular-niche astrocytes foster GCSs (95, 96). In addition, astrocytes in the PBZ express glial cell line-derived neurotrophic factor (GDNF) to promote invasive tumor growth (97).

As astrocytes cannot respond to inflammatory stimuli, one of the mechanisms for their activation is their interaction with microglia (98). Tenascin-C, osteopontin, lactadherin, and fibulin-3, which are secreted by tumor cells to promote tumor development and invasion, modify ECM and tumor microenvironment components, which in turn alter the complex microglia-astrocytes-tumor cell interactions (99). Reactive astrocytes build a thick network in the area of tumor invasion in the PBZ as a result of aberrant plasticity of the CNS and consequent alterations in the composition of the microenvironment (100). Reactive astrocytes secrete high levels of growth factors, cytokines, and other chemicals that are then exposed to the GB milieu, triggering various pathways to promote tumor growth. Released by astrocytes near the tumor, TNF-α, TGF-β, IGF-1, and VEGF enhance GB cell proliferation and invasion (101). Astrocytes support tumor growth and nutrient supply (102). Given that, it has been reported that reactive astrocyte depletion regresses GB and increases mice survival (103). In addition, IL-6 produced by astrocytes appears to participate in many processes that drive GB development, including promoting proliferation and invasion, as well as modulating the immune response to tumor progression (104, 105). Therefore, this important cytokine may be taken into account as a target for novel treatments. Therefore, focusing on particular environmental elements may enhance the effectiveness of immune therapies in addition to regular treatment with targeted immunotherapy alone.

Although reactive astrocytes are key players in promoting GB invasion, proliferation and resistance to therapy, they remain under-studied. Hence, the effectiveness of therapeutic approaches for GB may be increased by a deeper understanding of the pathogenic mechanisms that drive interactions between GB cells and reactive astrocytes in the PBZ.

### Endothelial cells in PBZ

Endothelial cells (ECs) in the brain are characterized by specific features and properties, given their role as the interface between the CNS and blood stream. ECs in the brain develop constant complexes of tight and adherent junctions between them, creating a tight and size-selective barrier. By controlling vascular permeability ECs are the primary cellular constituents of the blood-brain barrier (BBB) (106). However, the EC phenotypic and functional characteristics in GB differ from the ones in normal tissues. In GB context, ECs form a close connection with tumor cells to change tissue homeostasis towards a tumor-supporting microenvironment (107). A clear difference exists between normal and tumor ECs which present biomarkers such as vWF, CD31, and CD105 (107). Tumor ECs loose CD144 (VE-cadherin), a protein that is crucial for vascular integrity (108). Furthermore, it has been shown that almost all CD31- and CD34-positive ECs co-express VEGFR-1 and -2 within the tumor core of GB. In contrast, the majority of CD31 and CD34-positive ECs in the PBZ lacked VEGFR expression, expressing Nestin but not Factor VIII. In the PBZ, vessels that are CD31, CD34, and nestin-positive but lack VEGFRs and Factor VIII may be signs that tumor vasculature is starting to proliferate. Furthermore, low VEGFR expression in PBZ ECs appears to correlate with the therapeutic failure of VEGFR-targeting drugs, which could explain why this area is frequently the source of recurrence after surgical resection (30). In addition, Xie et al. discovered five distinct EC phenotypes that corresponded to different anatomical regions: the tumor core and PBZ, representing various degrees of ECs activity and BBB disruption. Using scRNA-seq, they discovered a cluster in the PBZ, characterized by elevated expression of genes typical of the BBB, such as KLF2 and SLC2A1. KLF2 is a crucial transcription factor that controls the gene networks that promote ECs quiescence. Furthermore, they discovered ECs with an angiogenic phenotype that expressed a high concentration of genes related to endothelial tip cells, angiogenesis, cytoskeleton rearrangement, and basement membrane remodelling. The authors identified CD93 as a critical regulator of the cytoskeleton and ECM organization in ECs during angiogenesis, collagen (COL4A1 and COL4A2), collagen-modifying enzyme (PXDN), and other basement membrane components (LAMB1 and HSPG2). In addition, numerous BBB transporters, including SLC2A1, ABCG2, ABCB1, SLCO1A2, and ATP10A, were overexpressed in the PBZ ECs compared to the tumor core (109).

### Spatial immune heterogeneity within PBZ

GB has been extensively characterized as an immune-cold tumor, and its immunosuppressive microenvironment promotes tumor development and growth by hampering an effective antitumor immune response (110). GB is supported by tumor-associated microglia and peripheral myeloid cells (111). In addition to CNS-resident microglia, which regulate homeostasis and safeguard the CNS against injury and infections (111), infiltrating immune cells in the GB include peripheral macrophages, granulocytes, neutrophils, myeloid-derived suppressor cells (MDSCs), and TILs (112), which infiltrate tumor tissue to different extents across the tumor core and PBZ.

### Tumor-associated macrophages and microglia within PBZ

TAMs, which make up to 40% of the tumor mass, are a combination of microglia and infiltrating macrophages, and represent an essential component of the tumor microenvironment. TAMs are crucial for the development, recurrence, and therapeutic response of tumors (113). Their differentiation has historically been based on different CD45 expression levels; while infiltrating macrophages show high levels of CD45 (CD11b+CD45^high^), microglia express low levels of CD45 (CD11b+CD45^low^). These include P2RY12, Sall1, Tmem119, and Hexb, which are expressed only by CNS-resident microglia (114–117), whereas CD49d and CXCR4 are preferentially expressed by infiltrating macrophages (118, 119). However, a comprehensive understanding of the spatial dynamics within the tumor core or PBZ and its clinical relevance has been poorly investigated. Data from a meta-analysis of single-cell and bulk RNA-seq of materials from patients with GB revealed that TAMs have a dynamic identity (with M0, M1, and M2 states). TAMs are more pro-inflammatory and have enhanced PD-1 signalling activity in the tumor core, while they are more anti-inflammatory and have higher NF-kB signalling in the PBZ (120). Based on this evidence, it has recently been shown that TAMs with anti-phagocytic actions express PD-1, which can be counteracted by PD-L1 blockage (121). Moreover, anti-PD-1 antibodies can directly activate macrophages, which leads to an increase in cytokine production, a pro-inflammatory phenotype, and improved tumor cell phagocytosis (122). Additionally, NF-κB has been shown to encourage polarization towards M1-like macrophages under normal conditions (123). However, in cancer, NF-κB signalling in tumor-associated macrophages inhibits M1 polarization and promotes M2 anti-inflammatory responses (124). Thus, inhibition of NF-κB signalling in myeloid cells promotes M1-like TAM polarisation in mice models of GB, resulting in increased cytotoxic T cell infiltration and decreased tumor growth (125, 126).

On the other hand, microglia have been reported to be predominant in the PBZ regions (127, 128). Conversely, Darmanis et al. highlighted that TAMs express more pro-inflammatory cytokine genes, such as IL1, in the PBZ than in the tumor core, where anti-inflammatory genes, such as TGFβ, are elevated (129). TGFβ expression by TAMs in the tumor core may be beneficial for tumor growth and dissemination, as TGFβ has been shown to inhibit cell adhesion, promote survival of cells with DNA damage, and act as a potential angiogenic factor. In contrast, the tumor core showed increased levels of IL1RN, a significant anti-inflammatory regulator that negatively interacts with IL1R1 to actively inhibit immune activation. These results have implications for therapeutic immunotherapy strategies against GB. However, these observations imply that the effectiveness of targeted immune therapies may significantly differ across different patients and that tumor phenotyping is essential for understanding the local distribution of immune cells and developing more effective and beneficial treatments. Moreover, according to Friebel et al., there is a subset of CD206^+^CD169^+^CD209^+^ macrophages in the tumor core that has been directly linked to blood vessels (130). Furthermore, Koshkaki et al. suggested that CD163+ TAMs are the most common cell type in both the PBZ and tumor core. Additionally, IDO and PDL-1 were nearly absent in the PBZ but were expressed in the majority of tumor core samples, frequently in conjunction with TIGIT expression. According to the authors, the significant immunosuppression seen in the GB microenvironment is a result of many inhibitory mechanisms that might work simultaneously in the tumor core and PBZ. Nevertheless, compared to the tumor core, PBZ exhibited significantly less immunosuppression (131). Nonetheless, unlike other neurodegenerative and inflammatory diseases, for which the different stages of activation and the different spatial distributions along different brain regions have been extensively described (117), the temporal and spatial dynamics of TAMs in brain tumors, as well as the functional differences among different subpopulations in the tumor core and PBZ are still largely unknown, and their clinical relevance remains poorly defined.

### Different T-cell subsets as key players across GB tumor tissue

The development of adaptive immunity against cancer is a self-replicating, cyclical process that begins with the antigens released by cancer cells and ends with the infiltration of activated effector T-cells (also known as TILs) into the tumor bed which recognize and targets tumor cells (132). Increased intratumoral effector cytotoxic and helper T-cell populations are substantially associated with improved survival (133). Nevertheless, as observed for TAMs, very few studies have compared the spatial and temporal differences in the different T cell types in the GB between the tumor core and PBZ. This aspect is crucial when considering T-cell exhaustion, where the tumor core is more prone to induce it and is characterized by decreased granzyme B, TNFα, IFNγ (134), and IL-2 (135) levels, thus impacting immunotherapy (136). Conversely, the PBZ contains a greater number of effector T-cells that are not exhausted (137). Moreover, the tumor core has more Foxp3^+^CD25^+^CD127^lo^CD4^+^ Tregs and myeloid-derived suppressor cells than the PBZ (138), indicating that the milieu in the core of the tumor is more immunosuppressive than the PBZ (137). Furthermore, it has been shown that tumor cores have more exhausted CD8^+^ T cells than the PBZ, and that hypoxia directly enhances CD8^+^ T-cell exhaustion (139). It was also discovered that a higher frequency of PD-1^+^CTLA-4^+^CD8^+^ T-cells in the tumor core than in the PBZ was strongly associated with a decreased PFS in patients with GB. CD4^+^ and NK cells are significantly higher in the tumor core than in the PBZ, whereas the number of dendritic cells is higher in the PBZ than in the core region (139). This study showed that immunosuppression and hypoxia were higher in the tumor core than in the PBZ. Moreover, a Phase I clinical trial assessed efficacy and feasibility of employing neural stem cells, already known to cross the BBB and infiltrate the tumor tissue (140), in order to delivery engineered oncolytic viruses in the PBZ. The treatment demonstrated that an antitumor response was associated with an increase in CD8+ T cells in the tumor (141).

Thus, based on evidence from the literature, it is possible to reconstruct a different spatial distribution between the tumor core and PBZ, which shows a more immunosuppressive and hypoxic environment in the tumor core together with a high number of Tregs, exhausted CD8^+^ T cells, and TAMs M2, which may suggest a much less susceptible environment for immunotherapy efficacy. Hence, there is an urgent need to better understand the spatial immune microenvironment in GB to develop novel immunotherapies for more efficacious treatments.

## Tissue mechanics in PBZ

Physical stress caused by GB plays a fundamental role in progression, immune evasion, and response to treatment, affecting both GB cells and the stroma of the PBZ. As GBs grow, they modify the structure of the brain and affect the function of the surrounding PBZ through several physical forces mechanisms, such as elevated solid stress, elevated interstitial fluid pressure (IFP), modified tissue stiffness and altered tissue microarchitecture (142).

### Interstitial fluid pressure in PBZ

IFP represents the isotropic stress exerted by the fluid phase. Stylianopoulos et al. discovered that the growth of tumors leads to an increase in fluid pressure, which is attributed to the increased permeability of blood vessels and the impairment of lymphatic function. This increase in fluid pressure is regulated by the pressure in the microvascular system. However, it is important to note that fluid pressure does not result in compression of tumor blood vessels (143). This presence of leaky blood vessels and a compromised drainage system in GB contributes to a high IFP of approximately 7.5 mmHg (142).

A high IFP hinders the convection of drugs from the vasculature into the tumor core and drives interstitial flow in the PBZ, exposing extravascular cells to shear stress (144). The pronounced IFP gradient in the PBZ propels the movement of interstitial fluid from the tumor core towards the PBZ. This fluid flow can promote tumor invasion and growth by facilitating the transport of released substances and GB cells to the PBZ.

### Solid stress in PBZ

The term “solid stress” refers to the pressure experienced by the solid components of tumors and the surrounding brain tissue, that is, the ECM and cells. IFP and solid stress are two separate types of mechanical stress, each with its own origin and impact on the tissue studied (142).

The elevated compressive solid stress within the tumor core leads to the collapse of blood vessels, which consequently results in a reduced growth rate of GB cells compared to those found in the periphery. Solid stress in the periphery of the GB causes blood vessels in the surrounding normal tissue to deform into elliptical shapes (143).

The growth pattern of the GB, which can be nodular or infiltrative, is a determining factor for intratumor and peritumor solid stress. Solid stresses are larger around more nodular GBs than around infiltrative GBs. Nodular GBs deform the surrounding PBZ and neuronal nuclei, thus reducing peritumor vascular perfusion and inducing neural loss (145). In addition, surgical resection modifies tissue mechanics in the PBZ adjacent to the resection cavity, which suddenly reduces solid stress and mechanical pressure, thus favoring GB cell proliferation towards the cavity, which is repopulated by GB cells over time (146).

### ECM stiffness in the tumor core and in the PBZ

GB develops within a microenvironment that is mechanically challenged and is characterized by a dense and stiff ECM, which leads to compromised vascular integrity and subsequently induces hypoxia (147).

Early investigations began to map and measure GB rigidity. However, disparities persist among research groups who emploied varying methodologies, such as magnetic resonance elastography (MRE) and atomic force microscopy (AFM), in both preclinical and clinical specimens.

The general consensus seems to be that the GB is stiffer than brain tissue. Additionally, it appeared that the GB stiffness in the tumor core was greater than that in the PBZ. However, it should be noted that the range of stiffness among the different regions of the GB is quite large, which suggests that there may be varying biological and mechanical features across different regions of the GB.

Stiffer environments may alter the GB cell biology. By employing a hydrogel platform with tunable stiffness that is degradable by matrix metalloproteinases for three-dimensional culture, it has been demonstrated that reducing the stiffness of the hydrogel enhances the proliferation of GB cells, whereas increasing the stiffness of the hydrogel enhances drug resistance in GB cells.

GB tissue stiffness may also modulate blood vessel perfusion. A prospective study aimed to map differences in biomechanical and functional properties between GB and healthy brain tissue, measuring the mechanical properties with MRE has been found to be a useful predictor of blood vessel perfusion. Specifically, tissue stiffness has been observed to be inversely correlated with the values obtained for perfusion (148).

In addition to a direct impact on blood perfusion and oxygen supply, mechanical changes and ECM produced by GB can also promote local immune dysfunction, by inhibiting T cell migration into the tumor core and PBZ (149). Furthermore, immune response and immunotherapy treatment effects not only require the generation of cancer-specific T-cells but also that these T-cells physically contact cancer cells (150).

All of the previously cited physical modifications favor the compression and disruption of blood and lymphatic vessels, hindering blood flow, supply of oxygen, proper functioning of immune cells, and impeding correct drug delivery. Therefore, a broader understanding of the relationship between the biology and physics of GB and its PBZ might provide opportunities for the discovery of new therapies.

Insights into tissue mechanics in the PBZ may offer a pivotal avenue for practical interventions to enhance the clinical outcomes in patients with GB. Understanding the role of elevated IFP and compressive solid stress within the tumor core and surrounding PBZ reveals critical factors influencing GB progression, treatment response, and immune evasion. ECM stiffness influences vascular integrity and induces hypoxia, thereby contributing to local immune dysfunction and hindering effective immunotherapy. Therefore, strategies focusing on modulating IFP, alleviating solid stress, and manipulating ECM stiffness present promising avenues for optimizing drug delivery, enhancing treatment response, and addressing immune-related challenges in GB patients, marking a significant step towards translating biomechanical knowledge into clinical advancements.

Importantly, to improve immunotherapy strategies, targeting and reducing IFP would promote better drug penetration and enhance immune cell access to the tumor core and PBZ. In the same way, strategies to alleviate compressive solid stress and modulate the stiff ECM may enhance immune cell mobility and functionality. Combining these tissue mechanics-targeted approaches with immunotherapies holds promise for synergistic benefits. Due to the mechanical heterogeneity of tissues among GB patients, it is important to think that personalized immunotherapeutic interventions adapted to individual biomechanical profiles should be considered.

## Clinical imaging

**Figure 2:**
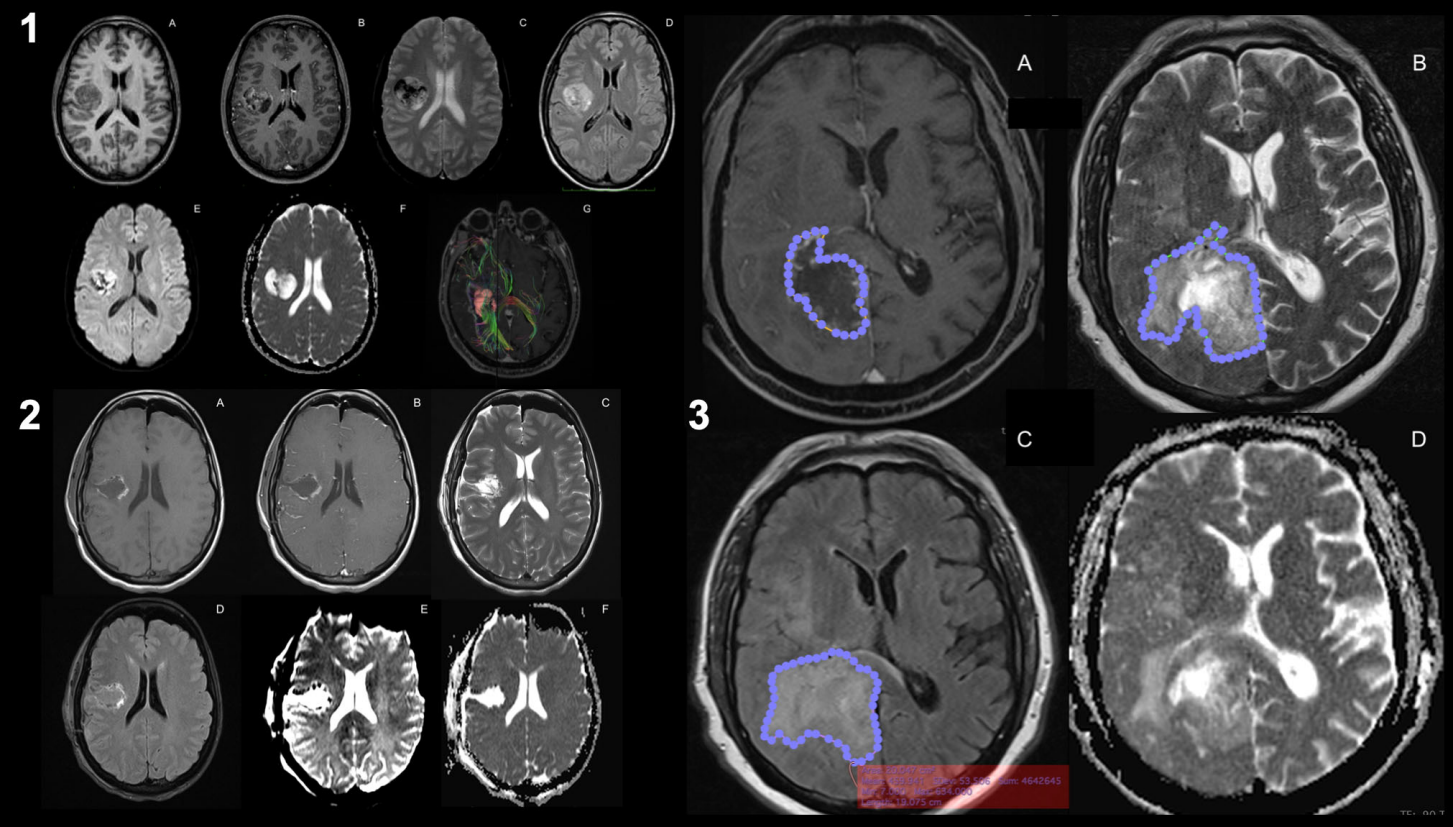
Magnetic resonance imaging of glioblastoma. Preoperative MRI-imaging sequences used for the study of glioblastoma (1): T1-weighted images (1.A), T1-weighted contrast-enhanced images (1.B), T2-weighted images (1.C), FLAIR images (1.D), perfusion imaging (PWI, 1.E), diffusion imaging (DWI, 1.F), and DTI with intra- and peri-lesional fiber-tracking study (1.G). Same sequences for the postoperative study of resection margins after the surgical procedure (2). Areas of the identified altered signals (3): the contrast-enhanced signal margin highlighted by a blue-dotted line (3.A), the signal margin in T2 associated with the presence of peri-lesional edema (3.B), the margin in the same lesion in FLAIR (3.C) and the DWI (3.D). It can be seen that there is a discrepancy between the signal area in FLAIR versus T2 (known as T2-FLAIR mismatch), which may imply that there are areas of PBZ within healthy edematous tissue.

GB growth is characterized by diffuse infiltration of normal brain tissue. However, techniques based on the identification of regions with disrupted BBB cannot accurately detect tumor infiltration beyond the apparent borders of the enhancing region (151, 152). Therefore, new MRI techniques may be useful for evaluating tumor cell infiltration in the PBZ; abnormal signal regions are frequently observed adjacent to or along the surgical cavity in clinical practice (8, 9, 18, 153). The main imaging techniques described in the literature are presented here (**Figure 2**): different contrast images in magnetic resonance imaging (MRI), such as T1- and T2-weighted imaging, FLAIR, diffusion-weighted imaging (DWI), apparent diffusion coefficient (ADC), diffusion tensor imaging (DTI), restriction spectrum imaging (RSI), and positron emission tomography and computed tomography (PET-CT) scans (**Supplementary Table 1**).

### T1-weighted-imaging

Contrast enhancement on standard brain MRI qualitatively reflects the disrupted state of the BBB. The PBZ displays a homogeneous dynamic contrast enhancement (DCE) profile, comparable to that of a healthy brain, but different from that of other GB sampling zones (14). This radiological profile is characterized by low permeability and low extracellular volume fraction that reflect the maintenance of the BBB in the PBZ, which would explain the absence of contrast enhancement (154). Tumor infiltration can occur in the absence of angiogenesis or vascular abnormalization. The tumor cell density threshold to modify the MRI signal is approximately 500 cells/mm^3^, which limits radiological examination of GB infiltration in the brain parenchyma. This was highlighted by the results of a histopathological examination that found GB cell infiltration in one-third of radiologically normal PBZ areas (14). Similarly, drug access to a viable contrast-enhanced tumor core is likely significantly higher than that of PBZ, which usually does not exhibit contrast enhancement (155).

### T2-weighted-imaging

Extensive edema in the PBZ is a characteristic feature of the GB. It is believed that when hyperintensity is found on T2 weighted imaging surrounding a tumor, it hosts infiltrating tumor cells (156). However, with this type of MRI sequence, it is currently impossible to distinguish edema related to the lesion from edema caused by GB cells. Furthermore, distinguishing among gliosis, radiation effects, tumor-containing tissue after recurrence, impaired blood flow due to injury to normal vessels and cytotoxic edema can be difficult (157).

### Combination of FLAIR and T2-weighted MRI

**Figure 3:**
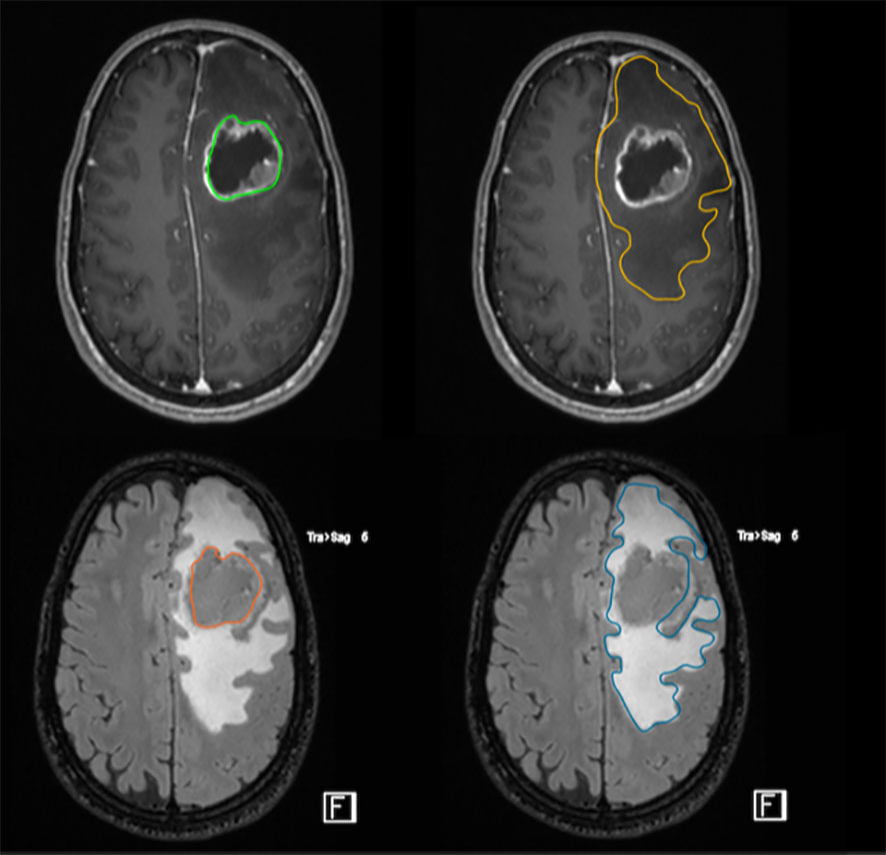
FLAIR-high lesions showing peritumoral brain zone in glioblastoma. The size of the peritumoral edema is one index to predict the degree of tumor infiltration through imaging. The surgical resection of FLAIR-high lesions is important to improve postoperative outcome.

The measurement of the residual T2/FLAIR abnormal signal region surrounding the surgical cavity defined as the postoperative peritumor edema zone (**Figure 3**), coupled with a high ratio of choline/N-acetyl-aspartate (Cho/NAA) ≥ 1.31 is associated with early recurrence and poor prognosis, proving this approach to be a useful prognostic tool to predict GB relapse (18).

On T2‐weighted (T2W) FLAIR, peritumoral hyperintensity usually fades toward the normal-appearing brain tissue, making it very difficult to identify a clear‐cut border. Some studies on MRI‐guided biopsies report features of diffusively infiltrating gliomas intermixed with apparently normal brain tissue on MRI. Thus, most imaging studies define the PBZ as the whole area of T2W hyperintensity surrounding the contrast-enhanced T1W brain tissue identified as the border of the tumor. A wide peritumoral T2W FLAIR hyperintensity is associated with elevated microvessel density and MIB‐1 index, whereas a narrow peritumoral FLAIR hyperintensity correlates with both low microvessel density and low MIB‐1 index. FLAIR signal could correspond to high CD163+ cells expression at bioptic analysis. CD163+ GB cell are involved in invasiveness, tumor cell migration, angiogenesis, immune evasion, edema formation, and a worse survival rate (158).

Moreover, the MIB-1 index (used as a complementary method to differentiate better and worse prognostic groups of astrocytic tumors) of the PBZ correlates with the degree of the FLAIR-high area. Low MIB-1 index in the PBZ correlated with narrow FLAIR-high areas, and high MIB-1 index in the PBZ correlated with broad FLAIR-high areas. In addition, the microvessel density (MVD) ratio of the PBZ to the tumor core also correlates with the degree of FLAIR-high areas; however, the association between postoperative edema and the prognosis of patients with GB is not well understood (159).

Importantly, patients with complete resection (>95%) who had greater than 53% of FLAIR abnormality resected in addition to the contrast-enhancing tumor showed improved survival compared with those who had less than 53% of FLAIR abnormalities resected (160, 161).

### Diffusion-weighted-imaging (DWI)

The apparent diffusion coefficient (ADC) derived from DWI, which represents the measurement of the magnitude of diffusion (of water molecules) within tissues, is inversely related to tissue cellularity and has been proposed as a noninvasive imaging biomarker for the detection of intratumor high-density areas. Postoperative edema associated with inflammation, infiltration, and necrosis increases extracellular diffusion. This increased diffusion may be a combined consequence of surgical injury, tumor cell invasion, or demyelination; however, these cannot be differentiated using routine MRI (18).

### Novel MRI techniques

Several studies have shown an association between preoperative GB MRI features and clinical prognosis, but few studies have focused on the imaging configuration of the PBZ. Moreover, radiological identification of the PBZ remains problematic. Indeed, the tumor boundary, which includes the necrotic area, can be easily identified by delineating the contrast enhancement area visible on post‐gadolinium T1‐weighted (T1W) images. Conversely, the no‐enhancing regions, beyond these limits, may show radiological features that are not specific to tumor tissue and may be indistinguishable from vasogenic edema and other non‐tumor tissue alterations. Multidirectional diffusion‐weighted imaging can be used to compute tensor‐based quantitative maps of water diffusion, such as apparent diffusion coefficient (ADC), fractional anisotropy, mean diffusivity, radial diffusivity, and axial diffusivity, all of which help distinguish vasogenic edema from tumor infiltration and high‐cellularity regions.

Novel diffusion-weighted MRI techniques, such as DTI and RSI (151) sequences, separate the relative contributions of hindered and restricted signals originating from the extracellular and intracellular water compartments. These techniques were used to assess PBZ infiltration and showed that vasogenic edema and tumor-infiltrated edema are characterized by distinct imaging patterns (152). They can be used to explore regional cerebral blood volume (CBV), cerebral blood flow, and vascular permeability indices, all of which are expected to be relatively higher in tumor areas associated with neoangiogenesis than in normal brain tissues. More recently, a relatively new functional MRI technique has been developed that allows the identification of brain areas associated with glycolytic metabolism leading to lactate production and pH reduction, which can be identified using MRI signal modifications induced by chemical exchange saturation transfer (CEST) resulting in acidity maps. Considering the highly hypoxic metabolism of GB, these MRI sequences may differentiate between IDH wild‐type and IDH‐mutant gliomas, which exhibit different pH distribution gradients (162). Therefore, these technologies can be used to differentiate peritumor edema from what could instead be the nest of a PBZ that is capable of generating recurrence (163). 1H magnetic resonance spectroscopy (1H-MRS) is widely used to non-invasively detect biochemical indices and metabolic changes in intracranial lesions, which can reinforce diagnostic confidence in distinguishing neoplastic and non-neoplastic intracranial lesions and distinguishing tumor recurrence from radiation necrosis (164).

Further improvements in the MRI diagnosis of the GB and its periphery are possible owing to active targeting. Specific targeting groups conjugated with contrast agents can improve recognition and accumulation within a particular tissue. For example, enhanced GB visualization has been observed for paramagnetic liposomes coupled with monoclonal antibodies (mAbs) against endoglin (CD105), a key protein involved in tumor angiogenesis. Under this aspect, radiomics could offer in coming future new tools for defining and identifying PBZ by capturing subtle quantitative measurements on MRI by computing local macro-and microscale morphological changes in texture patterns within the lesion. Peritumor radiomic features have already been found to be predictive of survival in GB across T2w and FLAIR sequences when compared with tumor enhancement and tumor necrosis features (165). The need for improved imaging is given by the importance of establishing proper preoperative planning to ensure total and supramarginal GB resection because it is known that tumor cells that have not yet developed a characteristic radiologic phenotype are present at the margins of the lesion and from the nests of cells (the PBZ) in more than 70% of recurrences.

Apart from radiomics studies, all other imaging techniques have entered the routine MRI protocols of primary brain tumors and are rapidly generating a huge amount of high‐definition structural and functional MRI multiparametric data, which can be correlated with histopathological, genetic, and molecular data. These types of analyses require high computing power and new imaging analysis methods to improve identification of the PBZ (166).

## Treating residual GB within the PBZ

**Figure 4:**
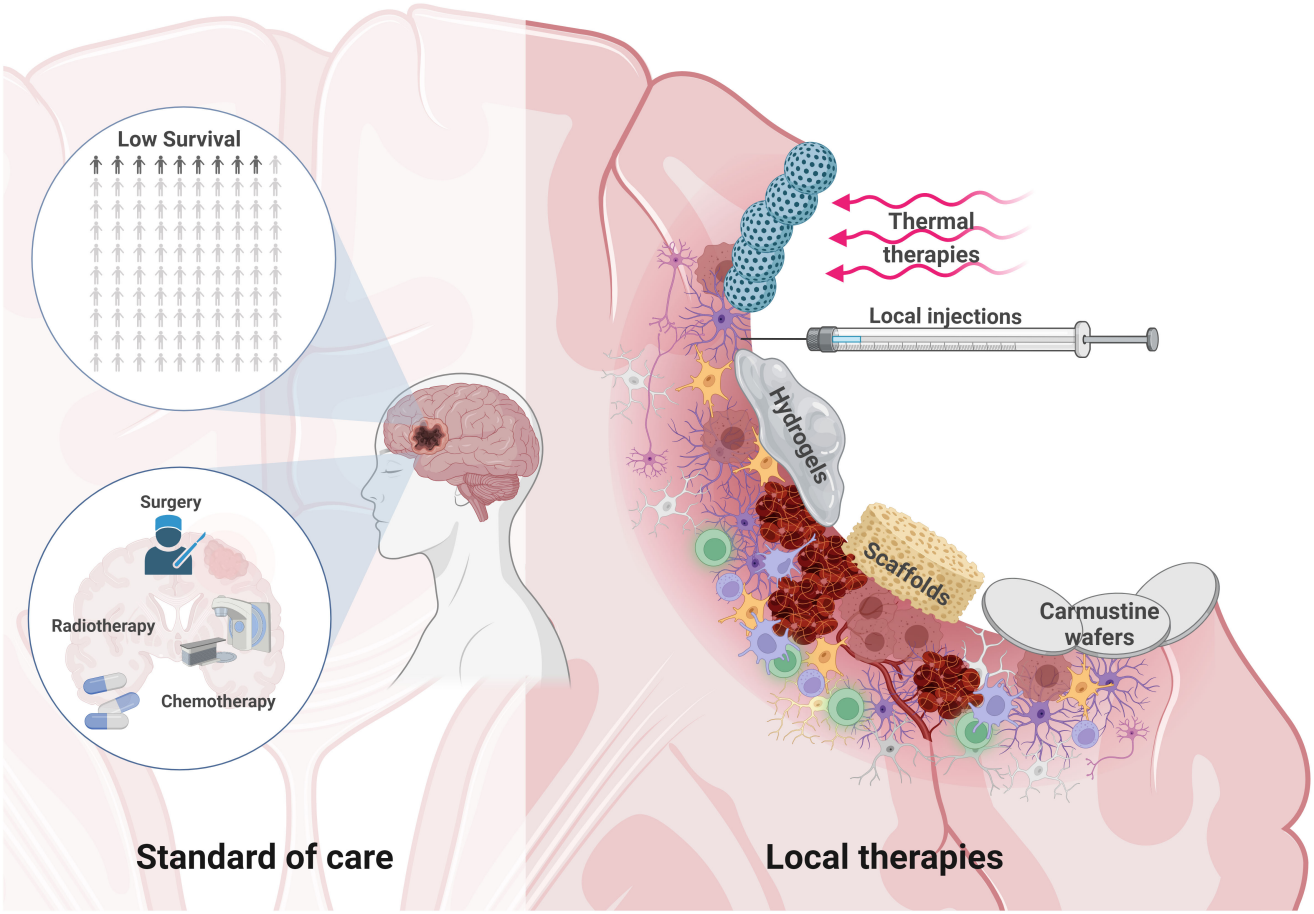
Local therapies to treat residual GB within the PBZ. The survival of glioblastoma patients is low despite the standard of care therapeutic approach based on microsurgical tumor resection, radiotherapy and chemotherapy (*Left*). Microsurgical resection opens a window of possibilities for local therapies. Brain injury can develop in the form of hematoma formation due to microbleeds, edema, neuroinflammation, and even hemorrhage. The PBZ is characterized by a heterogeneous and dynamic microenvironment where the interaction between the resident immune, vascular and cellular components takes place. Some local therapeutic approaches in this resected area are being studied, such as the use thermal therapies and nanoparticles that are stimulated by external magnetic fields, local injections, hydrogels, carmustine wafers or various scaffolds to deliver drugs locally, among other therapeutic approaches (*Right*). Created with www.BioRender.com.

Most GB recurrences are located near the resection cavity (167), to treat or prevent them, several local therapies have been developed and tested in preclinical and clinical trials. These therapeutic approaches aim to treat residual and infiltrating GB cells in the postoperative PBZ (**Figure 4**). These therapies also aim to avoid drug delivery problems in the tumor niche due to the BBB, vessel dysfunction, local fluid pressure, hematomas or hard-to-reach poorly perfused hypoxic areas. At the same time, these local approaches represent an opportunity, since aggressive treatments can be administered locally without significant risks of systemic toxicity.

Achieving precise drug delivery to the PBZ confronts multifaceted challenges in the complex tumor microenvironment. The heterogeneity of GB, marked by diverse cell types and genetic variations, demands nuanced strategies to ensure comprehensive coverage within the PBZ. BBB requires an inventive approach to facilitate optimal drug penetration. Tumor vasculature irregularities and perfusion variations introduce complexities in uniform drug distribution, whereas the infiltrative nature of GB mandates addressing the surrounding infiltrated regions. The immunosuppressive microenvironment of GB adds an additional layer of complexity, requiring interventions to overcome immune evasion for enhanced treatment efficacy. Challenges persist in accurately monitoring drug distribution in real time, limiting our ability to precisely track therapeutic agents within the PBZ. Furthermore, the spatial and temporal heterogeneity of the tumor microenvironment underscores the need for adaptable strategies to accommodate dynamic changes.

Several approaches have been explored for local delivery of drugs using hydrogels (168–170) and other drug-impregnated biodegradable polymers (171–173). Biodegradable carmustine wafers have been used to administer high doses of chemotherapy to the resection cavity (174, 175). Carmustine functions as an alkylating agent by forming interstrand crosslinks in the DNA, which prevents DNA replication and transcription. Its use in trials with patients has shown improvements in overall survival when used with the Stupp protocol (176); however, its use has been associated with adverse events, such as seizures, weakness on one side of the body, nausea, vomiting, and fever.

Implantable Ommaya reservoirs connected to the resection cavity and/or brain ventricles with a catheter have been used to deliver high doses of chemotherapy over time (177, 178). These reservoirs are implanted under the skin and can be accessed through brief interventions when the doctor considers them appropriate, allowing personalized adaptation of the patient’s treatment. However, to date, patient improvement in clinical trials has been minimal.

Direct intraoperative injections after surgical resection (179, 180) and stereotactic injections with the aid of neuronavigation coupled with computed tomography (CT) or MRI have been studied (181, 182); however, poor diffusion of compounds into the brain parenchyma has been described. Convection-enhanced delivery (CED) method has been explored to improve drug delivery (183, 184). This establishes a positive pressure gradient that improves the spatial distribution of treatment and can be left in place for a prolonged period of time. Nevertheless, the use of this method is challenging; precise cannula placement is crucial but difficult, and learning how to use it appropriately by surgeons requires a steep learning curve (185). In addition, the distribution of the infused treatment varies because the GB and PBZ are heterogeneous among patients, with different foci, degrees of necrosis, and variation in vascular anatomy and metabolism. Furthermore, it varies by area even within the same patient. Future optimization of CED may revolve around refining the cannula design, personalizing treatment plans, and adopting monitoring techniques with advanced imaging to track the therapeutic agent distribution, thereby minimizing variability and ensuring consistent outcomes.

Thermal therapies, such as laser interstitial thermal therapy (LITT) (155, 186–188), focused ultrasound (FUS) (189, 190), and magnetic hyperthermia (191, 192) have also been developed to treat residual GB cells in the surgical cavity because GB cells are sensitive to temperature, leading to apoptosis and/or necrosis.

LITT involves inserting an optical fiber into the tumor and heating it with laser light. The extent of tissue ablation during LITT is a critical factor that influences its efficacy. Aggressive treatment may lead to higher rates of adverse effects, including permanent neurological symptoms, cerebral edema, seizures, and hydrocephalus (193).

Under MRI guidance, transcranial FUS has emerged as a tool for transiently disrupting the BBB and improving drug delivery and penetration. However, its application in GB is limited, and ongoing trials are exploring its potential. The main limitations are attenuation owing to skull thickness and ablation of healthy tissue in the path of ultrasound waves (194).

Magnetic hyperthermia involves the generation of heat by magnetic nanoparticles. In addition to its direct effects, hyperthermia can sensitize GB cells to radiotherapy and chemotherapy (185, 195). Although slight improvements have been achieved in clinical trials using these thermal treatments, undesired side effects related to worsening of neurological symptoms in patients, with edema and seizures, have also been reported. Hence, further investigations are required, emphasizing the need for well-designed trials.

Photodynamic therapy (PDT) is a local therapeutic approach that uses photosensitizing agents in the resection cavity. After activation by light of a certain wavelength, they generate reactive oxygen species that cause extensive DNA damage, leading to cell death (196, 197). One of the main drawbacks of this therapy is that light must reach the sensitizing agent. However, with the wavelengths currently used, tissue penetration is minimal, typically a few millimeters, although some studies have attempted to improve penetration into brain tissues (198). Alternative light sources with deeper penetration capabilities and innovative delivery systems should be explored.

Recently, local viral gene therapies for GB have attracted significant interest. Notably, some patients exhibit impressive responses to these treatments. One extensively researched virus is the replication-incompetent adenoviral vector AdvHSV-tk, delivering the herpes simplex virus type 1 (HSV-1) thymidine kinase gene (199), that by administering an antiviral (like ganciclovir) leads to thymidine kinase phosphorylating the prodrug, causing it to bind to DNA during double-strand break repair, disrupting mitosis and DNA repair mechanisms, inducing cell apoptosis and improving sensitivity to chemoradiation. Recently, in a Phase III clinical trial, AdvHSV-tk under the control of the promoter of the early growth response gene 1 (EGR-1) was employed to intraoperative transduce GB cells by local administration in the PBZ area. Following surgical resection, patients were intravenously treated with ganciclovir to target the sensitive HSV-tk expressing GB cells (200). Furthermore, gene therapy-based immunotherapeutic strategies that harness the host immune system’s ability to specifically target and eliminate glioma cells, while also developing immunological memory have demonstrated significant progress. Two adenoviral vectors expressing Ad-HSV1-TK/GCV and Ad-Flt3L have shown promising preclinical data (201), which has led to FDA phase I human trial that have shown safety and feasibility in high-grade glioma patients (202).

Many of these local approaches have shown great potential for improving PFS and/or OS in several phase I and II trials; however, adverse effects have also been described, not yet allowing the translation of these promising results and the addition of these therapeutic tools to the standard treatment. However, promising results have been obtained, offering potential avenues to enhance patient outcomes. Innovative techniques, such as LITT, magnetic hyperthermia, FUS, PDT, and CED have demonstrated encouraging prospects. However, translating these promises into standard clinical practice requires addressing the key challenges. These include the need for conclusive clinical trials to validate the safety and efficacy of emerging modalities, refine precision in cannula placement and infusate distribution, overcome hurdles in implantable technologies, and establish standardized or individualized protocols. As we navigate through these challenges, the evolving landscape of local therapies for residual GB shows exciting potential, emphasizing the importance of continued research and strategic integration into routine clinical practice.

## Future perspectives and impact of PBZ research

The molecular and cellular heterogeneity of PBZ in GB presents a formidable challenge in the field of neuro-oncology. This heterogeneity extends beyond genetic variations to encompass diverse cellular phenotypes and distinct spatial microenvironments within tumors. The tumor microenvironment, intricately woven with the ECM, immune cells, and blood vessels, plays a crucial role in GB progression and therapeutic response. Tissue mechanics within the PBZ further contribute to this complexity, with solid stress, IFP, and ECM stiffness influencing tumor behavior. Understanding these mechanical aspects will open new avenues for novel therapeutic intervention. Cutting-edge imaging techniques have emerged as powerful tools to dissect this heterogeneity and explore the intricacies of the PBZ. However, there remains a need for more precise imaging methodologies that can capture dynamic changes within the PBZ and guide localized therapeutic strategies. Local therapeutic options (hydrogels, carmustine wafers, implantable reservoirs, CED, LITT, FUS, etc.) have been developed to address spatial and temporal heterogeneity within the infiltrative areas of GB, especially around the surgical resection cavity. As neuro-oncology navigates the intricate landscape of GB and its PBZ heterogeneity, the convergence of insights from tissue mechanics, advancements in imaging technologies, and the development of localized therapeutic modalities collectively define the trajectory toward precision medicine for individuals afflicted by this pathology.

## Author contributions

AB: Writing – original draft, Writing – review & editing. DA: Writing – original draft, Writing – review & editing. VR: Writing – original draft, Writing – review & editing. GS: Writing – original draft, Writing – review & editing.

## Supplementary material

Supplementary Table 1 Primary outcomes revealed by different imaging techniques and limitations in the identification of the peritumoral brain zone (PBZ).
