# Supplementary material for: Peritumoral brain zone in glioblastoma: biological, clinical and mechanical features
Source: Front Immunol. 2024 Feb 29;15:1347877. doi: 10.3389/fimmu.2024.1347877 (PMC10937439; doi:10.3389/fimmu.2024.1347877)
Supplement: Supplementary Table 1 — Primary outcomes revealed by different imaging techniques and limitations in the identification of the peritumoral brain zone (PBZ). [file Table_1.docx]

**Supplementary table 1.** Primary outcomes revealed by different imaging techniques and limitations in the identification of the peritumoral brain zone (PBZ).

| Radiological appearance of PBZ | | |
| --- | --- | --- |
|  | Sequences | PBZ area |
| CT scan |  | Signal not visible or covered by hyperdensity from brain edema |
| **MRI scan** | T1W | No signal |
|  | T2W | Signal covered by hyperhintensity from brain edema |
|  | T2W FLAIR | PBZ-Signal and brain edema |
|  | DWI ADC | PBZ-Signal and brain edema (high fractional anisotropy, mean diffusivity, radial diffusivity, axial diffusivity could help to distinguish it) |
|  | T1W CE | PBZ-Signal and brain edema |
| 18F‐FET PET |  | PBZ-Signal and brain edema, with hypermetabolism suggests active tumor infiltration |
